# Health status based on EQ-5D-5L for the cancer patient population in Malaysia

**DOI:** 10.1038/s41598-024-58844-8

**Published:** 2024-04-08

**Authors:** Sharifa Ezat Wan Puteh, Hasyimah Razali, Aniza Ismail, Malina Zulkifli

**Affiliations:** 1 Department of Public Health Medicine, Faculty of Medicine, Universiti Kebangsaan Malaysia, Jalan Yaacob Latif, Bandar Tun Razak, 56000 Cheras, Wilayah Persekutuan Kuala Lumpur, Malaysia; 2 Department of Management, Faculty of Business, Universiti Teknologi MARA (UiTM) Kedah Branches, Campus Sg. Petani, 08400 Merbok, Kedah, Malaysia; 3 Faculty of Public Health, Universitas Sumatera Utara, Jalan Universitas No. 21 Kampus USU, Medan, 20155 North Sumatra Indonesia; 4 School of Quantitative Sciences, Northern University of Malaysia, UUM Sintok, 06010 Kedah, Malaysia

**Keywords:** EQ-5D-5L, Index, EQ-VAS, Cancer, Health status, Quality of life, Health care, Cancer

## Abstract

The EQ-5D is a common generic tool used in clinical trials and economic evaluations to evaluate the health-related quality of life as a proxy of health outcomes. To date, studies using EQ-5D-5L to evaluate the health status of cancer patients remain scarce in Malaysia. In this study, EQ-5D-5L dimensions, EQ-5D-5L index, and EQ-VAS scores were applied to assess the health status of Malaysian cancer patients. A cross-sectional study was conducted March-December 2022 to collect data relevant to the EQ-5D-5L valuation of health status via the Research Electronic Data Capture (REDCap) platform. Respondents rated their health states using EQ-5D-5L and EQ-VAS. Among the 235 respondents, the mean EQ-5D-5L index and EQ-VAS score were 0.76 (SD 0.223) and 81.06 (SD 16.36). Most of the patients reported some problems in the pain/discomfort and anxiety/depression dimensions. The level of education, stage of cancer, and comorbidity were significantly associated with better health status on EQ-5D-5L (*p* < 0.05) but only the stage of cancer was significantly associated with EQ-VAS scores. This study highlighted the disparities in self-reported health status across patients of different sociodemographic and medical profiles with EQ-5D-5L valuation. Thus, future research should use EQ-5D norm scores as a benchmark of comparison among cancer patients.

## Introduction

Globally, cancer contributes to a high level of morbidity and mortality^1^. Over the last few decades, many steps have been taken to lower the disease burden of cancer. However, the incidence of cancer continues to increase^2^. In Malaysia, as high as 115,238 new cancer cases were reported between 2012 and 2016^3^. It also ranks as the second commonest cause of death. In 2016, breast cancer had the highest incidence rate in Malaysia at 17.3% followed by colorectal cancer at 13.6% and lung cancer at 8.7%^4^. These three types of cancer, make up half of all reported cancer cases in Malaysia. Unfortunately, these cancers are often diagnosed at an advanced stage.

The escalating cancer incidence has caused a great concern in the country, especially with the implications on the healthcare system and the quality of life (QoL) of affected patients as they require more medical attention^5^. The growing need for health economic evaluation arises due to scarcity of resources and escalating expenses associated with medical care especially for cancer diseases^6^. Cost utility analysis (CUA) is a favoured approach in health economic evaluation, wherein the measurement of health outcomes is conducted by utilizing quality-adjusted life years (QALYs)^7^. Measuring the health status of cancer patient is essential for effective treatment, disease monitoring, patient care, research, and policy-making, leading to improved outcomes and quality of life for individuals affected by cancer.

Various methods have been devised to measure health status, including direct and indirect methods^6,7^. Worldwide, EQ-5D is one of the most commonly applied patient-reported outcome measures (PROM) instruments in which the health state of an individual is classified using a descriptive system to facilitate the use of an indirect method to evaluate the health state^8,9^. In other words, the EQ-5D represents standardised preference-based generic health measures that are easy to use in clinical and economic assessment^10^.

EQ-5D is commonly applied in the evaluation of healthcare interventions and public health strategies as it is simple to use besides providing rich information as compared to other similar instruments^11^. In a multinational study with a large dataset, EQ-5D-5L was found to have higher accuracy. It was also preferable based on the study results and participants’ responses^12,13^. EQ-5D has been employed in numerous countries to assess the health of the general population, and norms specific to age, gender, and socio-economic status have been established^14,15^. However, there is a lack of established EQ-5D-5L norms for the cancer patient population in Malaysia.

In this paper, we set out to assess the health status for common cancer patients in Malaysia by using EQ-5D-5L dimensions, EQ-5D-5L index, and EQ-VAS scores. Moreover, this study also set out to identify any relationships between the sociodemographic and medical profiles of the respondents with their EQ-5D-5L index and EQ-VAS scores.

## Methods

### Study design

This cross-sectional survey study was conducted with the REDCap (Research Electronic Data Capture) software hosted at Universiti Sains Malaysia (USM) for data collection between March and December 2022^16,17^. REDCap is a secure and web-based software platform that supports data capture for research studies. The role of the software includes: (1) to provide an intuitive interface to validate data capture; (2) audit trails to track data manipulation and export procedures; (3) perform automated export procedures for seamless data downloads to common statistical packages, and; (4) carry out procedures for data integration and interoperability with external sources.

### Sampling and recruitment

To establish a population norm, the data related the individual’s health state and demographic information were collected from the respondents. Based on the formula by Bhalerao and Kadam^18^, the calculated sample size was 235 respondents. The study sample included patients with breast, colon, and lung cancers. These three cancers reported the highest number of new cases each year based on the Malaysia National Cancer Registry Report (MNCRR) 2012–2016^3^. The respondents were purposively sampled through the official pages of the Colorectal Cancer Association, Cancer Survivor Malaysia, Breast Cancer Welfare Association Malaysia, and Cancer Fighter Malaysia on a social media platform. Purposive sampling was used to ensure that the study population would fulfil the objectives of this systematic research by capturing the characteristics of numerous subgroups. Adult patients (at least 18 years old) living in Malaysia who were diagnosed with breast, colon, or lung cancer, who could communicate in the Malay language, and who voluntarily agreed to participate were included. On the contrary, patients diagnosed with other cancers, Malay illiterate, and unable to complete an online questionnaire were excluded from this study.

### Sociodemographic and medical profile characteristics

Sociodemographic characteristics included age, gender, race, marital status, education level, monthly household income, employment, and insurance status while medical profiles such as types of cancer, stage of cancer, and comorbidity were collected for analysis.

### EQ-5D-5L dimensions

In the EQ-5D-5L, respondents will score the five dimensions of health (mobility, self-care, usual activities, pain/discomfort, and anxiety/depression) on five levels of severity, ranging from 1 = "no problems", 2 = "slight problems", 3 = “moderate problems”, 4 = “severe problems”, to 5 = “extreme problems”. By combining the level of each dimension, a unique state of health can be obtained. For the coding purpose, each health level is referenced by five digits. For example, code 12345 indicates no problem with mobility, slight problems with washing or dressing, moderate problems with usual activities, severe problems with pain or discomfort, and lastly, extreme problems with anxiety or depression. In contrast, code 11111 represents a lack of problems in all dimensions^10^. Between 11111 (no problems on any dimension) and 55555 (extreme problems or unable to on all dimensions), EQ-5D-5L can be used to describe a total of 3125 health states^19^.

### EQ-5D-5L index

To obtain the EQ-5D summary index, a value (weight) is attached to each level in each dimension. The index is then calculated by subtracting the appropriate weight from 0 to 1, as 1 represents a full health status. The collection of index values (weights) for all possible EQ-5D health states will give rise to the value set. Most sets of EQ-5D values were generated from standardised assessment exercises whereby a study sample from the general population of a country or region was invited to rate their EQ-5D health states. In 2019, the EuroQol Group established a standardised assessment study protocol (EQ-VT) to produce a set of standard values for the EQ-5D-5L among different study populations. This protocol was developed based on the composite time trade-off evaluation technique (cTTO) and discrete choice experiment (DCE) to simplify the evaluation technique, and more importantly, to facilitate international comparisons^9^. The index utility value is attached to the responses on the EQ-5D-5L descriptive system based on the specific tariff of the Malaysian population^20^. The utility index value can be obtained from the evaluation of the health condition by the general population and it is a good proxy of the patient's health condition from the general population’s perspective. When computed, the EQ-5D-5L index scores will range from a health status rated worse than death (0) to full health (1).

### EQ-VAS scores

By using EQ-VAS, the overall health condition from the patient’s perspective on the day of answering the questionnaire can be elicited. It is scored on a scale ranging from 0 (worst imaginable state of health) to 100 (best imaginable state of health).

### Data analysis

SPSS for Windows (version 28.0) was used for data analysis. The health status of each respondent were directly retrieved from the self-report questionnaires of EQ-5D-5L and EQ-VAS. The Malaysia EQ-5D-5L value set was then used to calculate the EQ index score^20^. All 3125 health states' EQ-5D-5L index scores were estimated after acquiring the EQ-5D-5L value set^19^. Each respondent's EQ-5D-5L index scores were calculated based on their self-reported health conditions.

Following that, descriptive summary statistics of EQ-5D-5L dimensions, EQ-5D-5L index, and EQ-VAS score were calculated for all the samples based on their sociodemographic characteristic (age, gender, race, married, education, household income, employment, and insurance) and medical profiles (types of cancer, stage of cancer, and comorbidity). The percentage of problems reported under the EQ-5D-5L dimensions, the means (95% confidence interval) of the EQ-5D-5L index, and EQ-VAS scores were determined for each subgroup for each sociodemographic variable. Secondly, Mann–Whitney U and Kruskal Wallis test was used to examine the significant difference between sociodemographic and medical profile characteristics with EQ-5D-5L dimensions, EQ-5D-5L index, and EQ VAS scores. The test was used as the data not normally distributed.

Lastly, multivariable analysis was conducted for the EQ-5D-5L index and EQ VAS score to analyse the relationships between multiple independent and dependent variables. The Universiti Kebangsaan Malaysia (UKM) Research Ethics Committee gave its clearance for this work, and it was conducted in accordance with UKM's established standards for ethical research. Study was performed according to the declaration of Helsinki. Prior to participating in the study, each patient gave their informed consent, which the institution’s ethics committee had approved.

### Ethical approval and consent to participate

This study has been approved by the Research Ethics Committee of Universiti Kebangsaan Malaysia (UKM) and the research ID was JEP-2020-504. Informed consent was obtained from all the participants.

## Results

There were 235 individuals in the final analysis. Among the 377 respondents who tapped the survey link on the REDCap software, 142 respondents (37.7%) with incomplete questionnaire were excluded. Table 1 shows the sociodemographic and medical profile characteristics. The mean age was 43 years (range 22–76 years old). The majority of them were females (77.9%), Malays (80.9%), and married (76.6%). A small number of patients (7.7%) had a Master's degree or Ph.D. education level while the rest had a Diploma (24.3%) or Degree (26.4%). Most of them had a monthly household income of below RM 3000 (46.8%), 25.5% earned RM 3001-RM 5000, 12.8% earned RM 5001-RM7000, and 14.9% earned RM 7001 and above.

**Table 1:** Sociodemographic and profile medical characteristics of respondents (N = 235).

Characteristics	Frequency	%
Age group, years		
18–34	48	20.4
35–44	88	37.4
45–59	88	37.4
60 above	11	4.7
Gender		
Male	52	22.1
Female	183	77.9
Race		
Malay	190	80.9
Chinese	23	9.8
India	12	5.1
Others	10	4.3
Married status		
Single	40	17.0
Married	180	76.6
Others	15	6.4
Level education		
Diploma	57	24.3
Degree	62	26.4
Master/PhD	18	7.7
Others	98	41.7
Household income		
RM0–RM3000	110	46.8
RM3001–RM5000	60	25.5
RM5001–RM7000	30	12.8
RM7001 above	35	14.9
Employment status		
Government	51	21.7
Private	62	26.4
Self-employed	48	20.4
Unemployed	74	31.5
Insurance status		
Have insurance	84	35.7
No insurance	151	64.3
Types of cancer		
Breast	143	60.9
Lung	29	12.3
Colorectal	63	26.8
Stage of cancer		
Stage I	47	20.0
Stage II	75	31.9
Stage III	68	28.9
Stage IV	45	19.1
Comorbidity		
Yes	88	37.4
No	147	62.6

From the results, a total of 60 types of respondents' health statuses were reported among the respondents. The highest health status was ‘11111’ (18.3%), followed by ‘11222’, ‘11122’, ‘11121’, and ‘11112’ respectively. The percentages of ‘no problems’ differed for each dimension, ranging from as high as 81.3% for self-care, 61.7% for mobility, 46.8% for usual activity, 38.3% for anxiety/depression, and lastly 27.7% for pain/discomfort. However, none of the respondents experienced extreme problems in the dimensions of mobility, self-care, and pain/discomfort (Table 2). Table 3 shows the mean EQ-5D-5L index was 0.77 (SD = 0.22; 95% CI 0.73,0.91) with values ranging from − 0.22 to 1 while the mean EQ-VAS score was 81.1 (SD = 16.36; 95% CI 78.96, 83.17). There was a statistically significant strong positive correlation between the EQ-5D-5L index and EQ-VAS scores (rho = 0.60, *p* < 0.000).

**Table 2:** Percentage of respondents for levels 1–5 by dimension.

	Mobility	Self-care	Usual activity	Pain/discomfort	Anxiety/depression
No problems	61.7	81.3	46.8	27.7	38.3
Slight problems	23.8	11.9	34.9	43.4	38.7
Moderate problems	11.5	5.1	12.3	21.7	12.8
Severe problems	3.0	1.7	3.4	7.2	6.8
Extreme problems	0	0	2.6	0	3.4

**Table 3:** The central tendency and dispersion of EQ-5D-5L index and EQ-VAS scores.

	EQ-5D-5L Index	EQ-VAS scores
Mean	0.7674	81.06
Median	0.799	85
SD	0.2233	16.36
Minimum	− 0.222	15
Maximum	1	100
95% CI (Lower–Upper)	0.73–0.79	78.96–83.17

Table 4 shows the distribution of reported problems in various severity levels and dimensions, as well as the mean (SD) of EQ-VAS and EQ index scores by age group. Based on the results, age group was significantly associated with the dimensions of mobility and usual activity with p values 0.010 and 0.033 respectively. In contrast, the EQ-VAS and EQ index scores of self-care, pain/discomfort, and anxiety/depression dimensions were not associated with the age group. Patients 60 years and above reported the highest mean EQ-VAS value (85.73, SD = 11.8) while the highest mean EQ-5D-5L index score was observed among patients 35–44 years old (0.79, SD = 0.235).

**Table 4:** Percentage of cancer patient population sample reporting levels 1 to 5 dimensions, EQ-5D-5L indexes, and EQ-VAS score by age group.

EQ-5D dimension		Age groups				Total
		18–34	35–44	45–59	60 + +	
		N = 48	N = 88	N = 88	N = 11	N = 235
Mobility	No problems	72.9	67.0	51.1	54.5	61.7
	Slight problems	18.7	18.2	31.8	27.3	23.8
	Moderate problems	4.2	12.5	14.8	9.1	11.5
	Severe problems	4.2	2.3	2.3	9.1	2.9
	Unable to	0	0	0	0	0
	*p* value				0.169 **(0.010)**	
Self-care	No problems	85.4	84.1	75.0	90.9	81.3
	Slight problems	10.4	8.0	18.2	0	11.9
	Moderate problems	2.1	5.7	6.8	0	5.1
	Severe problems	2.1	2.2	0	9.1	1.7
	Unable to	0	0	0	0	0
	*p* value				0.078 (0.231)	
Usual activity	No problems	52.1	54.5	35.2	54.5	46.8
	Slight problems	35.4	29.9	42.0	18.2	34.9
	Moderate problems	8.3	9.0	17.0	18.2	12.3
	Severe problems	4.2	3.3	3.4	0.0	3.4
	Unable to	0.0	3.3	2.4	9.1	2.6
	*p* value				0.139 **(0.033)**	
Pain/discomfort	No problems	18.8	34.1	26.1	27.3	27.6
	Slight problems	45.8	37.5	47.7	45.4	43.4
	Moderate problems	29.1	21.6	17.0	27.3	21.7
	Severe problems	6.3	6.8	9.2	0.0	7.3
	Unable to	0.0	0	0	0.0	0
	*p* value				− 0.048 (0.460)	
Anxiety/depression	No problems	18.8	45.5	37.5	72.8	38.3
	Slight problems	56.2	33.0	37.5	18.1	38.7
	Moderate problems	14.5	12.5	13.6	0.0	12.8
	Severe problems	4.1	4.5	10.2	9.1	6.8
	Unable to	6.4	4.5	1.2	0	3.4
	*p* value				− 0.114 (0.082)	
EQ-VAS	Mean	78.48	82.73	80.23	85.73	81.06
	SD	19.8	15.9	15.1	11.8	16.4
	95% CI	72.7	79.3	77.0	77.8	78.9
		84.2	86.1	83.4	93.6	83.1
	*p* value				− 0.010 (0.878	
EQ Index score	Mean	0.761	0.788	0.747	0.787	0.767
	SD	0.220	0.235	0.212	0.228	0.223
	95% CI	0.696	0.738	0.702	0.634	0.738
		0.825	0.838	0.792	0.940	0.796
	*p* value				− 0.024 (0.716)	

Significant values are in bold.

The association between EQ-5D-5L dimensions, indexes, and EQ VAS scores with the independent variables is outlined in Table 5. Based on the analysis, the level of education was significantly correlated with all dimensions of EQ-5D-5L. Meanwhile, the stage of cancer, and comorbidity were also significantly correlated with all dimensions of EQ-5D-5L, except for the anxiety/depression dimension. In contrast, there was no significant correlation between marital status and insurance ownership with any outcome variables. However, age, gender, education, type of cancer, stage of cancer, and comorbidity showed a significant difference with the mobility dimension *p*-value < 0.05). More importantly, EQ-5D-5L indexes and EQ-VAS scores showed significant correlations with monthly household income, stage of cancer, and comorbidity.

**Table 5:** Association of sociodemographic and medical profile characteristics with EQ-5D-5L dimension, indexes and EQ-VAS (N = 235).

Variables	Mobility	Self-care	Usual activity	Pain/discomfort	Anxiety/depression	EQ5D index	EQ VAS
*z/χ*^*2*^* (p value)*							
Age^1^	− 2.526 (0.012)*	− 1.398 (0.162)	− 2.468 (0.014)*	− 0.121 (0.904)	− 0.562 (0.574)	− 1.386 (0.166)	− 0.884 (0.377)
Gender^1^	− 2.999 (0.003)*	− 2.505 (0.012)*	− 2.032 (0.042)*	− 1.814 (0.070)	− 2.641 (0.008)*	− 2.821 (0.005)*	− 0.318 (0.751)
Race^1^	− 1.388 (0.165)	− 2.630 (0.009)*	− 2.588 (0.010)*	− 3.485 (0.000)*	3.286 (0.001)*	− 3.109 (0.002)*	− 0.237 (0.813)
Marital^1^	− 0.097 (0.922)	− 1.962 (0.050)	− 0.213 (0.831)	− 0.431 (0.666)	− 1.563 (0.118)	− 0.950 (0.342)	− 0.150 (0.881)
Education^1^	− 2.440 (0.015)*	− 3.387 (0.001)*	− 3.778 (0.000)*	− 3.571 (0.000)*	− 2.754 (0.006)*	− 3.413 **(0.001)**	− 0.794 (0.427)
Monthly household income^1^	− 1.726 (0.084)	− 1.211 (0.226)	− 2.792 (0.005)*	− 3.753 (0.000)*	− 2.420 (0.016)*	− 3.365 (0.001)*	− 2.131 (0.033)*
Employment^1^	− 0.937 (0.349)	− 2.243 (0.025)*	− 1.936 (0.053)	− 0.465 (0.642)	− 1.072 (0.284)	− 0.892 (0.372)	− 2.075 (0.038)*
Insurance^1^	− 0.062 (0.950)	− 1.027 (0.304)	− 1.262 (0.207)	− 0.814 (0.415)	− 1.063 (0.288)	− 1.113 (0.266)	− 1.638 (0.101)
Types of cancer^2^	9.667 (0.008)*	12.605 (0.002)*	2.306 (0.316)	3.715 (0.156)	9.218 (0.010)*	7.100 (0.029)*	0.017 (0.992)
Stage of cancer^2^	27.787 (0.000)*	12.287 (0.006)*	13.334 (0.004)*	16.298 (0.001)*	3.039 (0.386)	16.667 (0.001)*	28.142 (0.000)*
Comorbidity^1^	− 3.575 (0.000)*	− 2.970 (0.003)*	− 3.122 (0.002)*	− 3.116 (0.002)*	− 1.356 (0.175)	− 3.317 (0.001)*	− 2.158 (0.031)*

*Correlation is significant at the 0.05 level (2-tailed).

1Mann Whitney U test.

2Kruskal Wallis test.

Significant values are in bold.

All the significant variables in univariate analysis were inserted into the multivariate linear regression model to identify the significant predictors of EQ-5D-5L indexes and EQ VAS scores (Table 6). A backward selection procedure to remove covariate with *p* > 0.05 was applied. Based on the non-standardised regression coefficient (B), education levels (B = 0.097; *p* = 0.002), the stage of cancer (B = − 0.068; *p* = 0.000), and comorbidity (B = 0.102; *p* = 0.000) were significantly associated with EQ-5D-5L index. Based on the adjusted R-squared value, all of the independent variables explained 25.7% of the influence of the factors on health status by EQ-5D-5L index. Meanwhile, the stage of cancer was the only significant predictor of EQ-VAS scores (B = − 4.988; *p* = 0.000). The adjusted R-squared value for EQ VAS was 13.4%. All the factors were not significant predictors of EQ-VAS scores, except for the stage of cancer.

**Table 6:** Multivariable regression models for sociodemographic and medical profiles with EQ-5D-5L index and EQ VAS scores.

Variables (N = 235)	EQ-5D-5L index (R^2^ = 0.257)			EQ VAS scores (R^2^ = 0.133)		
	B	SE	*p* value	B	SE	*p* value
Age	0.030	0.30	0.319	3.125	2.871	0.278
Gender	0.080	0.044	0.072	3.364	4.177	0.422
Race	-0.031	0.049	0.528	0.008	4.636	0.999
Marital	0.006	0.040	0.882	3.149	3.840	0.413
Education	0.06	0.032	**0.003**	3.800	3.031	0.212
Monthly household income	0.047	0.032	0.148	4.783	3.083	0.123
Employment	− 0.021	0.033	0.524	− 0.826	3.114	0.791
Insurance	0.013	0.032	0.684	− 0.423	3.014	0.889
Types of cancer	0.015	0.021	0.466	2.612	1.969	0.186
Stage of cancer	− 0.039	0.014	**0.008**	− 4.634	1.371	**< 0.001**
Comorbidity	0.064	0.031	**0.042**	5.289	2.984	0.078

Bold values are significant at *p* value < 0.05.

SE standard error.

## Discussion

Based on literature review, there are very few studies in Malaysia that have reported EQ-5D-5L for patients with primary cancers in Malaysia^21–23^. The health status of the cancer population was obtained using EQ-5D-5L dimensions, EQ-5D-5L indexes, and EQ VAS scores. These findings represent vital insights into the HRQoL of cancer patients in Malaysia, specifically on the variation of HRQoL among different sociodemographic groups. Most significantly, it facilitates the conduct of cost effectiveness studies that incorporate QALY as a health outcome. By having a set of norm scores as a point of reference, the HRQoL of respondents obtained from any studies can be compared with the general population, especially among individuals with particular diagnosis which are of similar ages^24^.

Overall, the mean EQ-5D-5L index and EQ-VAS scores of cancer patients in this study were 0.76 and 81.00, respectively. Their mean EQ-VAS score was lower (85.52, SD 12.3)^20^ while the median EQ-5D-5L index (0.79) was higher than the general Malaysian population (0.336). Furthermore, the mean values of the EQ-5D-5L index were higher than two published studies that reported the utility values of patients with colorectal cancer based on EQ-5D-5L in Iran and China^6,11,25^. The difference in values could be attributed to the variation in the populations sampled, the value sets employed, and also the versions of the EQ-5D used in the survey^26^. Moreover, the Chinese EQ-5D-5L tariff was derived from the preferences of the Chinese and Iranian general public, both of which could be different due to cultural and demographic variations. Similar differences were observed when comparing with Asian countries such as Thailand (EQ-5D-5L index 0.90, EQ-VAS score 80.00)^27^.

Next, the study results revealed that most of the cancer patients did not experience any problem with mobility, self-care, and usual activity. In addition, no patient reported extreme problems in the dimensions of mobility, self-care, and pain/discomfort, in contrast to the dimensions of usual activity and anxiety/ depression. These results were in line with the findings in other countries whereby the first three dimensions of EQ-5D were commonly associated with fewer reported problems compared to the last two dimensions^15,26,28–33^. Based on previous studies, the percentage of reported problems in the dimension of anxiety/depression increased with age^28–30,34^. However, the opposite finding was shown in our results in which anxiety/depression problem was more prevalent in the younger population. Generally, there was a high prevalence of patients aged 35–60 years and above 60 years with no reported problems in the dimension of anxiety/depression. According to a study in China, the difference in age values could be explained by the psychological pressures experienced by younger generation compared to older patients^15^. In addition, no significant difference has been found between marital and insurance status across all dimensions of EQ-5D-5L. Previous reports have emphasized equal impacts on quality of life across different marital statuses^22,35^. Meanwhile, the lack of impact of insurance status on cancer patients may be attributed to Malaysia's status as an upper-middle-income country that provides subsidized public health services to all citizens through national taxation or lack of knowledge regarding health insurance^36^.

Apart from that, we also assessed if the patient’s sociodemographic and medical profile characteristics were associated with EQ-5D-5L index and EQ-VAS scores. The current study demonstrated that education level, the stage of cancer, and comorbidity were significant predictors of the EQ-5D-5L index. However, only the stage of cancer was a significant predictor of EQ-VAS. Moreover, adult patients were more likely to have more comorbidities and advanced stage of cancer, thus further limiting their physical functions and compromising their HRQoL. This result was in contrast with a previous study in which comorbidities did not show any significant influence on health utility^22^. This could be explained by the different types and number of comorbidities that might affect people’s perceptions and preferences during health state valuations^8^. Our results concurred with another study that reported education as an important factor that influenced health utility^11^. A high education level improved the patient’s perceptions of their disease and treatment whereas a low level of education can result in a delay in treatment-seeking^25^. However, in a Canadian EQ-5D-5L valuation paper, educational level was not linked to the value of health states^8^. A plausible explanation for this could be the underlying differences in the socioeconomic status, as well as the structure and accessibility of healthcare systems between both countries, hence influencing the correlation between educational level and health states^1,37^.

This study has several limitations. Firstly, only patients from the three most common types of cancers were included, thus limiting the generalisability of the results. Secondly, the Covid-19 pandemic necessitated the study to be undertaken fully on the digital platform, thus no direct interaction and face-to-face meetings with respondents were possible during data collection. Consequently, certain respondents without social media accounts or direct internet access could not participate in the survey. Previous studies reported that online surveys tend to have a lower response rate^38–40^, and incentives such as consolation money can sometimes improve the response rate^41^. Furthermore, response rates are typically lower among racial and ethnic minority groups, as well as those with poorer health status, lower income, and less education^42^. Additionally, a study found that breast cancer patients who were older, with a lower level of education, and a poorer quality of life preferred paper-based surveys compared to electronic surveys, thus indicating the potential barrier of data collection in this population^43^.

## Conclusions

Health state utilities is a highly potential tool to measure the effectiveness of treatment modality as determined by the treatment outcome by assessing the life expectancy of cancer patients based on their QALY. This study provided vital information about the EQ-5D-5L for the top three common types of cancers (breast, colon, and lung) in the Malaysian population. The findings highlighted disparities across sociodemographic groups in the self-reported health status measured by EQ-5D-5L. Therefore, healthcare providers and researchers should focus on the assessment of patients’ individual experience of disease and preference for treatment.

## Data Availability

The datasets used and/or analysed during the current study are available from the corresponding author on reasonable request.

## Abbreviations

EQ-5D-5L: EuroQol five dimensions five level
PROM: Patient-reported outcome measures
REDCap: Research electronic data capture
MNCRR: Malaysia national cancer registry report
QOL: Quality of life
HRQoL: Health related quality of life
CUA: Cost utility analysis
QALY: Quality adjusted life years
